# Effects of Different Cow-Milk Beta-Caseins on the Gut–Brain Axis: A Narrative Review of Preclinical, Animal, and Human Studies

**DOI:** 10.1093/nutrit/nuae099

**Published:** 2024-07-18

**Authors:** Stephen R Robinson, Frank L Greenway, Richard C Deth, Flavia Fayet-Moore

**Affiliations:** School of Health and Biomedical Sciences, RMIT University, Bundoora, 3083 Victoria, Australia; Pennington Biomedical Research Center, Louisiana State University System, Baton Rouge, LA 70808, United States; Department of Pharmaceutical Sciences, Nova Southeastern University, Fort Lauderdale, FL 33328, United States; Department of Science, FOODiQ, New South Wales, Sydney, Australia; School of Environmental and Life Sciences, The University of Newcastle, Ourimbah, 2258 New South Wales, Australia

**Keywords:** beta-casomorphins, dairy, inflammation, oxidative stress, aromatic amino acids

## Abstract

The gut and brain communicate through bidirectional neural, endocrine, and immune signals to coordinate central nervous system activity with gastrointestinal function. Dysregulated inflammation can promote immune cell activation and increase entero-endocrine signaling and intestinal permeability; hence, a functional gut–brain axis is necessary for a healthy digestive system. The consumption of milk products can lead to gut discomfort via effects on gastrointestinal tract function and the inflammatory state, which, in turn, affect the brain. A1 β-casein and A2 β-casein are major components of bovine-milk protein, and their digestion may result in different physiological effects following the consumption of milk products. Peptides derived from A1 β-casein, such as β-casomorphins, may increase gut dysfunction and inflammation, thereby modulating the availability of bioactive metabolites in the bloodstream and contribute to changes in cognitive function. This narrative review examines the functional interrelationships between the consumption of cow-milk–derived β-caseins and their effect on the brain, immune system, and the gut, which together comprise the gut–brain axis.

## INTRODUCTION

### The gut–brain axis

Miscommunication in the bidirectional communication network between the enteric and central nervous systems, termed the gut–brain axis (GBA), is now understood to underpin the etiology of many disorders of the central nervous system (CNS) and the gastrointestinal (GI) tract. There is a growing body of evidence indicating that immune modulation and inflammation are key factors that influence GBA communication.^1^^,^^2^ For instance, conditions associated with intestinal inflammation, such as irritable bowel syndrome and inflammatory bowel disease,^2^^,^^3^ are bidirectionally linked to CNS disorders, including depression, anxiety, and autism spectrum disorder (ASD).^4–6^ The gut is linked to the CNS via the vagus nerve, a major branch of the parasympathetic nervous system that transmits neuroimmune signals.^7^^,^^8^ Altered or disrupted vagus nerve activity is associated with gut inflammation and dysfunction.^7^^,^^9^ Dysregulated inflammation promotes immune cell activation, affecting both the CNS and the GI tract.^1^^,^^2^ Additionally, healthy functioning of the immune system is facilitated by compounds with antioxidant activity, such as certain vitamins and minerals, and glutathione, which can be influenced by the health of the GI tract and dietary intake.^10^^,^^11^ Diet influences the composition of the gut microbiota, which generates a variety of bioactive metabolites that can enter the bloodstream to modulate stress response and behavior at the level of the CNS.^12^^,^^13^ The release of bioactive molecules during digestion influences neural, endocrine, and immune pathways. These studies highlight the importance of the GBA in maintaining both gut and brain health.

### Milk and the gut–brain axis

Milk products comprise a significant portion of the diet in many populations globally and have several beneficial effects on health.^14–16^ For instance, bovine milk (hereafter referred to as milk) is highly nutritious^17^ and provides a large contribution to the global intake of vitamins A, B_2_, B_5_, and B_12_; phosphorus; potassium; and calcium.^18^ Although half of the world’s population may not receive sufficient calcium in their diets,^19^ some people avoid consuming milk and milk-derived products as they can be associated with symptoms of gut discomfort.^20^ Globally, a large percentage of the world’s population is thought to be intolerant to lactose, which can result in significant gut discomfort following the consumption of milk and other milk products.^21^ Moreover, the literature published over the past decade indicates that peptides released by the digestion of milk proteins (caseins, whey protein, and albumin) can exert a wide range of physiological effects.^22^^,^^23^ Milk proteins can influence GI digestion, absorption of metabolites, and inflammatory status, which, in turn, can affect the cardiovascular system and CNS via the GBA.^22^ Therefore, the digestion and absorption of milk, milk-derived products, and their components are important for understanding how diet may influence the GBA.

As the literature on milk proteins and their effects on health is expanding, this narrative review examines the functional interrelationships between the consumption of milk-derived proteins, including β-caseins, their effects on the GBA, and their mechanisms of action, highlighting areas for further research.

## METHODS

A comprehensive literature search of original articles published since 2000 was conducted using the PubMed, Web of Science, and Google Scholar databases. Searches used combinations of the following terms: “bovine milk,” “beta-casein,” “bowel inflammation,” “inflammatory bowel disease,” “beta-casomorphin,” “BCM-7,” “μ-opioid receptor,” “dipeptidyl peptidase-IV,” “lactose intolerance,” “glutathione,” “short chain fatty acids,” “gut microbiome,” “epigenetic,” “brain,” and “cognition.” The search results were screened for inclusion by examining the titles and abstracts. Articles not written in English were excluded (with 1 exception), as were articles that were determined to have low relevance. Where relevant papers addressed similar topics, preference was generally given to the more recent paper. The full texts of publications eligible for inclusion in the review were carefully reviewed.

## MILK PROTEIN DIGESTION

### β-Casein

Approximately 80% of the protein in milk is casein, which exists in 4 isoforms. The most common form of casein in milk is β-casein (25%–35% of total casein), of which A1 β-casein and A2 β-casein are the most common variants, differing from each other by only a single amino acid.^24^ There are 12 recognized genetic variants of β-casein, but they can generally be viewed as A1- or A2-type based on this difference between the 2 most common variants.^24^ The vast majority of commercially produced milk and milk products contain both A1-type β-casein and A2-type β-casein, whereas milk that is naturally devoid of A1-type β-caseins (A1 β-casein–free milk) accounts for only a small percentage of the world market.

**Figure 1.**
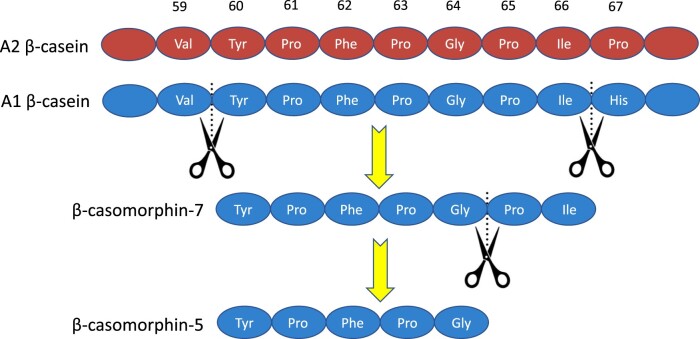
Cleavage of β-Casomorphin-7 from A1 β-Casein. In A2 β-casein, the proline at position 67 forms a bond with isoleucine at position 66 that is relatively resistant to cleavage by proteases. The histidine substitution in A1 β-casein enables this bond to be cleaved more easily (scissor icons), yielding β-casomorphin-7, which can be degraded further to β-casomorphin-5. Modified from Pal et al^25^

From an evolutionary standpoint, A2 β-casein is the original β-casein that is dominant in the milk of most ruminants. A point mutation in the A2 β-casein gene led to the replacement of a proline residue at position 67 in the polypeptide chain with a histidine residue, forming A1 β-casein (Figure 1^25^). Both A1 β-casein and A2 β-casein are found in milk from many breeds of cattle of European origin, whereas A2 β-casein is the predominant type of β-casein in milk from purebred Asian and African cattle.^26–29^

### β-Casomorphins and μ-opioid receptors

Casein aggregates into quaternary structures with calcium, known as micelles, which then self-assemble into insoluble curds that are slowly digested in the gut lumen.^30–32^ One of the most bioactive groups of peptides released by β-casein digestion in the gut lumen are the β-casomorphins (BCMs). The BCMs are smaller fragments (5 or 7 amino-acid peptides) that can bind to μ-opioid receptors within the intestine and enter the bloodstream. A1-type and A2-type β-caseins have a differential susceptibility to cleave into BCMs because of their different molecular structures (Figure 1^25^). The histidine substitution at position 67 (replacing a proline residue) changes its ability to form polyproline-II secondary structures, which may contribute to the development of larger micelles,^32^ and renders A1-type β-casein more vulnerable to cleavage by proteolytic enzymes in the gut than A2-type β-casein.^33^ Enzymatic digestion of β-casein results in a 7-amino-acid peptide (fragment 60–66), BCM-7, which is a strong agonist of μ-opioid receptors.^25^ BCM-7 can be subsequently cleaved to a 5-amino-acid peptide (fragment 60–64), BCM-5.^34^

μ-Opioid receptors are abundantly expressed by neurons of the enteric nervous system that supply the intestinal wall,^35^^,^^36^ and activation of these receptors regulates aspects of GI function, including motility and increased mucus and hormone secretion (such as somatostatin).^37–40^ For instance, BCM-7 reduces the frequency and amplitude of intestinal contractions, slowing the transit of food through the GI tract.^41^^,^^42^

**Figure 2.**
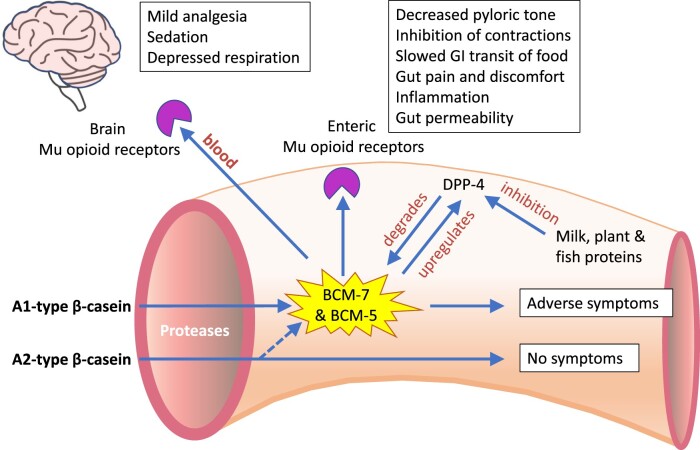
Factors Regulating the Production and Degradation of β-Casomorphin-7 (BCM-7) from A1-Type β-Casein in the Jejunum, and the Main Physiological Effects of β-Casomorphin-7 on the Enteric and Central Nervous Systems. The dotted line from A2-type β-casein indicates that the generation of β-casomorphins is possible, but it occurs at a slow rate. Abbreviations: BCM-5, β-casomorphin-5; GI, gastrointestinal; DPP-4, dipeptidyl peptidase-IV

The degradation of BCM-7 primarily occurs via the action of dipeptidyl peptidase IV (DPP-4) (Figure 2), an enzyme expressed by several cell types, including enterocytes, lymphocytes, endothelial cells, and neurons in the brain; a soluble form of DPP-4 is found in plasma and other body fluids.^43^ Most notably, DPP-4 is strongly expressed in the cells of the brush-border membrane of the small intestine and is present on exosomes secreted into the lumen, yet it is absent from the colon.^43^ The availability and enzymatic activity of DPP-4 largely influence the clearance rate of BCM-7 from the small intestine. Barnett et al^44^ demonstrated that the activity of DPP-4 in the jejunum of adult rats fed A1 β-casein was 40% higher than in rats fed A2 β-casein, suggesting that the gut is capable of a compensatory upregulation of DPP-4 in the presence of increased titers of BCM-7 (Figure 2). However, many other proteins and peptides are able to potently inhibit the activity of DPP-4, such as food-protein hydrolysates derived from milk, plants, and fish.^45^ It is possible that the presence of the protein hydrolysates may counter any compensatory upregulation of DPP-4 (Figure 2). Additionally, single nucleotide polymorphisms in DPP-4 have been associated with responsiveness to drugs that target DPP-4, and with the risk of developing certain health conditions, including heart failure, type 2 diabetes mellitus, and dyslipidemia.^46–52^ Furthermore, the effects of these polymorphisms may vary between males and females.^53^ It is possible that polymorphisms in DPP-4 may result in changes to the metabolism of BCM-7, and screening for these polymorphisms should be considered in studies evaluating the effects of A1 β-casein and BCM-7. Further research is required to fully understand how components of the diet and differences in DPP-4 interact to increase or decrease the half-life of BCM-7 in the gut.

In addition to cells of the enteric nervous system, μ-opioid receptors are located in the CNS, throughout the cortical and subcortical regions, including the homeostatic centers of the brainstem and hypothalamus.^54^ Importantly, BCM-7 and BCM-5 are able to cross the blood–brain barrier and bind to CNS μ-opioid receptors, and like other exogenous opioids, can produce mild analgesic effects, sedation, and respiratory depression (Figure 2).^55–57^ Moreover, research indicates that opioid receptors and their ligands play a role in immune-mediated GI pathophysiology.^58^^,^^59^

### BCM-7 and inflammation/oxidation

Several reports have indicated that BCM-7 can influence cellular antioxidant defenses via glutathione, a potent antioxidant via cysteine uptake. For instance, μ-opioid receptor agonists, including BCM-7, can inhibit cysteine uptake in human neural and GI epithelial cells, which is also associated with lower levels of intracellular glutathione.^60^^,^^61^ The ratio between glutathione and its oxidized form, glutathione disulfide, is indicative of cellular redox status and oxidative stress.^62^^,^^63^ Many neurological disorders are associated with decreased levels of glutathione in the brain and blood. These include Alzheimer’s disease, amyotrophic lateral sclerosis, ASD, bipolar disorder, Parkinson’s disease, and schizophrenia.^60^^,^^64^^,^^65^ Interestingly, healthy participants who consumed A1 β-casein–free milk showed increased blood levels of glutathione compared with those consuming milk containing both A1-type and A2-type β-casein (conventional milk),^66^ which could be due to the higher propensity of A1 β-casein to be cleaved to generate BCM-7.^66^ Similar results were found in a study of preschool children in China.^67^ Additional studies investigating the relationship between milk composition, cysteine uptake, and glutathione levels are warranted, particularly with regard to their antioxidant effects.

### ΒCM-7 and inflammation via epigenetics

Epigenetics describes the chemical modulation of DNA in response to environmental changes. During these changes, methyl and acetyl groups are added or removed from DNA to regulate gene expression.^10^ In addition to the biosynthesis of glutathione, μ-opioid receptor agonists can influence the production of *S*-adenosyl methionine (SAMe), a methyl donor that is important for the epigenetic modulation of DNA. The levels of SAMe are determined by cellular cysteine uptake via the ability of cysteine to modulate redox status. This affects the enzymatic activity of methionine synthase, which, in turn, is responsible for SAMe synthesis.^60^ This relationship underlies the hypothesis that opioid agonists, including food-derived opioid peptides, are able to influence gene expression through their indirect effect on SAMe levels.^60^ The use of SAMe has shown promising results for the treatment of depression and other CNS disorders, highlighting the potential therapeutic effects of targeting this pathway.^68^^,^^69^

Milk casein–derived peptides were found to increase DNA methylation of transcriptional start sites in human neural and GI epithelial cells in vitro, leading to changes in the expression of genes involved in redox and methylation processes, as described earlier.^60^ In fact, BCM-7 also altered the expression of several genes that are linked to gut inflammation and GI disease.^61^ It stands to reason, therefore, that since DNA methylation is crucial during developmental stages, particularly in the brain, the putative association between gut inflammation and neurodevelopmental disorders such as ASD and attention-deficit/hyperactivity disorder^4^^,^^70^ may be, in part, due to epigenetic changes affecting the expression of genes linked to inflammation. However, the importance of this mechanism for development and neural function remains to be determined.

### Neurological effects of BCM-7

BCM-7 can cross the blood–brain barrier,^55^ where it is able to act on cells of the CNS. In vitro differentiation of neural stem cells was found to be influenced by the addition of 1 µM BCM-7 for 4 hours.^61^ This effect was suggested to be related to epigenetic changes associated with genes related to neurogenesis. Although this mechanism remains to be investigated more thoroughly, studies in rodents indicate that BCM-7 can influence learning behaviors in neonatal rat pups.^71^ Neonatal rat pups injected intraperitoneally with BCM-7 daily over postnatal days 1–14 demonstrated impaired active and passive avoidance conditioning in a learning task 3–4 weeks post-birth, whereas in T-maze experiments involving food reinforcement, BCM-7 accelerated learning compared with placebo-injected pups.^71^ It was speculated that the anxiolytic effects of BCM-7 may have improved the rate of learning in the maze task, whereas the combined anxiolytic and analgesic effects of BCM-7 hindered avoidance conditioning. The effects on learning persisted up to 4 weeks after the last dose of BCM-7, suggesting that BCM-7 has longer term effects and that fear and motivation pathways in the brain may have been changed; however, it is unclear how long these effects on learning and brain development persist.

### β-Casein consumption and cognitive function in humans

Despite the evidence in rodent models that A1 β-casein affects the GBA and that administration of BCM-7 affects behavior,^55^^,^^72^ few studies have directly examined the influence of BCM-7 on learning and cognition in humans. There is some emerging evidence that the composition of A1 and A2 β-caseins in milk may influence cognitive function in humans, including individuals with lactose intolerance (defined as those who lack lactase activity in the gut).^73^

**Figure 3.**
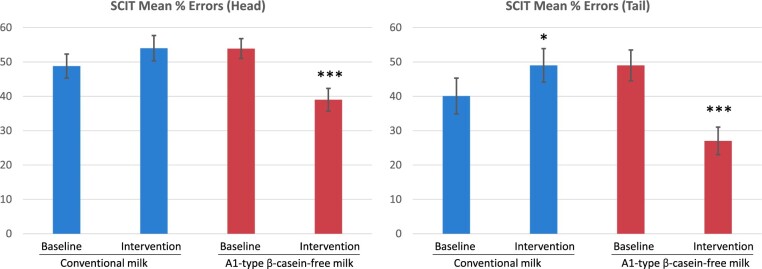
Performance on the Subtle Cognitive Impairment Test (SCIT) Following Consumption of Conventional Milk (Milk Containing Both A1 and A2 β-Casein) or Milk Containing Only A2 β-Casein (A1-Type β-Casein–Free Milk). The mean values for head errors and tail errors (expressed as a percentage of the number of total responses) are shown at baseline (after washout) and following twice-daily consumption of milk for 5 days. Error bars indicate the 95% CI of the mean. *P < .05 (baseline vs intervention; 2-tailed *t* test); ***P < .001 (baseline vs intervention; 2-tailed *t* test). The data have been replotted from Sheng et al^67^

In the study by Sheng et al^67^ lactose-intolerant preschool children who received twice-daily A1-type β-casein–free milk had improvements in error rates for “head” and “tail” on the Subtle Cognitive Impairment Test (SCIT) when compared with baseline performance, whereas “tail” errors increased significantly after consuming conventional milk (Figure 3). The “head” corresponds to subconscious information processing, while the “tail” corresponds to conscious decision-making, suggesting that the β-casein composition of milk may influence multiple aspects of cognitive function. Jianqin et al^74^ tested 45 adults on the SCIT at 4 time points: (1) at the end of the baseline washout period (between switching from conventional milk to A1 β-casein–free milk or vice versa), (2) after 2 weeks of daily consumption of 1 of the milks, (3) at the end of the washout period, and (4) after 2 weeks of daily consumption of the second milk. Compared with baseline scores, there were small, but significant, increases in both response time and error rate following consumption of conventional milk, whereas there were no changes after consuming A1 β-casein–free milk. Further, serum inflammatory markers were elevated among participants consuming the conventional milk, which may be linked to these changes in the cognitive function observed.^74^ While these findings support the conclusion that A1 β-casein–free milk is beneficial for cognitive function in adults, the mechanisms are unknown. It is possible that the benefits are related to increased levels of short-chain fatty acids (SCFAs) (further described later), which were observed following the consumption of A1 β-casein–free milk when compared with the consumption of conventional milk.^67^ It is possible that the type of β-casein, and consequently BCM-7, may influence changes in cognitive function and performance; however, the relevance of these effects in modulating cognitive function and learning in a real-world setting remains to be determined.

### Effects of BCM-7 on immune cell function: preclinical and animal evidence

Inflammatory conditions of the GI tract are associated with disorders of the CNS, and vice versa.^4–6^ Hence, a better understanding of the link between immune function, gut health, and the CNS is of importance. In the gut, β-casein and its derivatives are known to interact with the immune system. BCM compounds have been shown to interact with the mucosal immune response by inhibiting lamina propria lymphocyte proliferation in an in vitro model.^75^ This effect was suggested to be mediated via BCM activity at opioid receptors. Conversely, chronic morphine treatment in humans is associated with gut barrier dysfunction, which promotes systemic inflammation,^76^ indicating that it is important to differentiate between acute and chronic activity of opioid agonists. BCM-7, via its opioid receptor agonist activity, can also affect mucin production by intestinal cells by modulating the expression of mucin genes, an effect that is reduced with the addition of an opioid receptor antagonist in an in vitro setting.^77^ The enhanced production of mucus by BCM-7 may promote innate immune function; however, if BCM-7 reaches cells of the respiratory tract, excess mucus production may lead to complications such as asthma and sinusitis,^77^ although there is a paucity of evidence showing a causal relationship in humans.^78–80^ In a mouse model of colorectal cancer, BCM-7 was shown to regulate immune cell differentiation, which may promote antitumor effects.^81^

Differential compositions of caseins in milk may exert distinct effects on gut inflammation. For instance, gut immune phenotyping of the small intestine in BALB/c mice fed a diet supplemented with either conventional milk or A1 β-casein–free milk demonstrated an increase in the percentage of natural killer cells within the small intestine compared with the control group that received no milk.^82^ However, only the conventional-milk group showed increased numbers of γ/δ T lymphocytes, suggesting increased inflammation, although the study’s authors found no other evidence for this. Conversely, mice fed a diet containing A1 β-casein–free milk demonstrated several marked changes in immune cell composition in the lumen of the small intestine, including increased T-helper CD4+ and B CD19+ lymphocytes compared with the other groups, while the proinflammatory T-cytotoxic CD8+ T lymphocytes were significantly reduced.^82^ Similar findings were found in mice supplemented with BCM-7 and BCM-5, which displayed elevated inflammatory molecules (eg, interleukin-4, histamine) and increased lymphocyte infiltration and expression of Toll-like receptors in the gut.^11^ These findings were associated with significant changes to the intestinal wall structure, in which the duodenum villi of the A1 β-casein–free milk group showed improved tropism compared with the conventional-milk group.^82^ Consistent with these findings, Wistar rats fed a milk-based diet containing A1 β-casein showed a 65% increase in myeloperoxidase activity, a marker of inflammation, compared with rats fed a milk-based diet containing A2 β-casein.^44^ In summary, studies in animals have demonstrated that the composition of β-caseins in milk may be of particular importance for gut inflammation, and may influence symptoms of GI discomfort.

### Effects of β-casein on immune cell function: human evidence

The composition of β-caseins in milk may also influence the immune response following ingestion in humans. Effective functioning of the immune system is closely linked to antioxidant status, with glutathione being essential for the actions of antigen-presenting cells, and for the proliferation of T lymphocytes in response to pathogens.^83^ In the study by Sheng et al^67^ children with mild-to-moderate lactose intolerance who consumed conventional milk for 5 days also had increased levels of circulating inflammatory biomarkers, such as interleukin-4 and several immunoglobins, compared with baseline. In contrast, no differences were found among children consuming A1-type β-casein–free milk.^67^ Serum glutathione concentrations relative to baseline were 1.6-fold higher in the group that consumed conventional milk, and were 2.1-fold higher in the group that consumed A1-type β-casein–free milk.^67^

**Figure 4.**
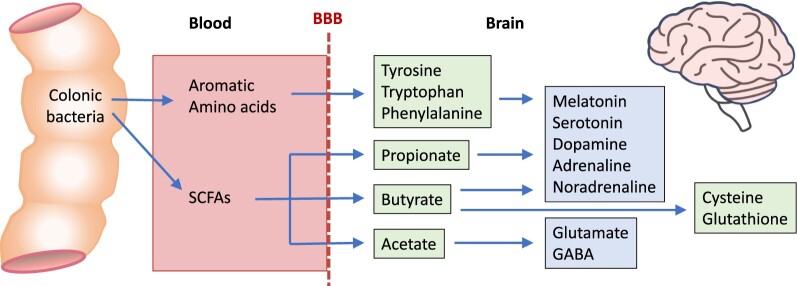
Essential Metabolites Synthesized by Colonic Bacteria That Are Required for Brain Function. The aromatic amino acids and short-chain fatty acids (SCFAs) are derived from the fermentation of proteins and peptides, such as β-casein and its breakdown products. The aromatic amino acids and SCFAs are transported from the colon into the blood and then across the blood–brain barrier (BBB) into the brain, where they serve as precursors or cofactors for the synthesis of the catecholamine neurotransmitters and 2 other important neurotransmitters (glutamate and γ-aminobutyric acid [GABA]). Butyrate is also a prerequisite for the synthesis of the antioxidants cysteine and glutathione

Studies by Deth et al^66^ and Jianqin et al^74^ reported that consumption of conventional milk was associated with a doubling of plasma glutathione concentrations relative to baseline, whereas consumption of A1 β-casein–free milk was correlated with an increase in glutathione by approximately 4-fold, suggesting that A2 β-caseins may confer greater antioxidant potential. Although the reason for this increase is not yet known, it may be speculated that increases in SCFAs, such as butyrate, play a role. SCFAs, particularly acetate and butyrate, have been shown to protect cells from reactive oxygen species and oxidative stress.^84^ Furthermore, butyrate upregulates the activity of several enzymes involved in the recycling of glutathione, as well as increasing the availability of glutathione (Figure 4).^84^ The contribution of BCM-7 and other BCMs derived from milk to immune function and the antioxidant response in humans remains to be determined.

### Effects of β-casein on gut health: human evidence

Recent findings indicate that milk-related GI discomfort may be influenced not only by the presence of lactose among those who are intolerant^21^ but by the composition of the β-caseins present. In a double-blind, crossover study, Ho et al^85^ compared outcomes following the consumption of conventional milk and A1 β-casein–free milk (both of which contain lactose), and demonstrated that abdominal pain and stool consistency measured by the Bristol Stool Scale were significantly increased in healthy and self-reported lactose-intolerant participants who consumed conventional milk, compared with those who consumed A1 β-casein–free milk. A similar crossover study found that both lactose absorbers and lactose malabsorbers experienced a higher frequency of intestinal symptoms, such as bloating, stool frequency, and abdominal pain, following a single meal of conventional milk versus A1 β-casein–free milk.^86^ Other studies of lactose-intolerant individuals and/or lactose malabsorbers have reported that the consumption of conventional milk was associated with the development of GI symptoms such as bloating, flatulence, borborygmus, nausea, and fecal urgency, while the consumption of A1 β-casein–free milk either significantly reduced or did not produce these effects.^67^^,^^74^^,^^87^ Interestingly, the consumption of A1 β-casein–free milk was associated with lower hydrogen breath production compared with conventional milk in a mixed group of lactose-intolerant and lactose-maldigesters, suggesting better lactose tolerance following the consumption of A1 β-casein–free milk.^88^ An explanation for this may be that some lactose-intolerant individuals are intolerant to A1 β-casein found in conventional milk and the associated effects of BCM-7.^25^ Conversely, Milan et al^87^ found that females without lactose malabsorption but with self-reported lactose intolerance experienced symptoms of digestive discomfort, such as nausea and bloating, in response to the consumption of conventional milk, lactose-free milk, and A1 β-casein–free milk, suggesting that there may be other compounds in milk that induce GI symptoms in some individuals. Other potential differences between the participants, such as polymorphisms in DPP-4^46–53^ or lactase,^89^^,^^90^ may also have contributed to the variability in reported GI symptoms. Therefore, future studies evaluating the effects of milk products on GI symptoms may benefit from including screening for these polymorphisms.

In summary, the composition of β-caseins in milk and milk-derived products appears to have a significant influence on GI symptoms following consumption. Those who experience lactose intolerance or discomfort following the ingestion of milk products may benefit from A1 β-casein–free milk.

### β-Caseins and the role of the gut microbiota in the gut–brain axis

In the average human adult, more than 100 trillion bacteria reside in the intestinal tract, which is 10 times the number of somatic cells in the human body.^91^^,^^92^ In recent decades, the importance of the gut microbiota for human health has become increasingly clear. For instance, gut bacteria are known to influence and interact with the host’s immune system, and a dysfunctional gut microbiota has been linked to inflammatory bowel disease, Crohn’s disease, ulcerative colitis, and colon cancer.^93^^,^^94^ Moreover, the gut microbiota has been linked to disorders of the CNS, including Alzheimer’s disease, Parkinson’s disease, depression, anxiety, multiple sclerosis, and ASD.^95–99^ The gut microbiota releases a wide range of biologically active metabolites, including neurotransmitters, which not only play a role in brain development but can also affect appetite, mood, sleep, the immune system, and the stress response.^100–102^ Hence, the composition of the gut microbiota is considered to be highly significant for the GBA.

Although the majority of proteins are degraded into amino acids and then absorbed by the epithelium of the small intestine, some proteins and peptides reach the colon.^103^ Gut microbiota preferentially ferment carbohydrates, such as dietary fiber and resistant starch, and switch to proteins and peptides once carbohydrates have been depleted. Consequently, carbohydrates are mostly fermented in the proximal colon, whereas proteins and peptides are fermented in the distal colon.^86^

If GI transit times are slowed, such as occurs in the presence of BCM-7, peptides are more likely to be degraded into amino acids before reaching the distal colon. Although all of the amino acids required by the human body can be obtained from the breakdown of β-casein, the 3 aromatic amino acids (phenylalanine, tryptophan, and tyrosine) can only be produced by the bacterial degradation of peptides using the shikimate biochemical pathway, which is lacking in human cells.^104^ Since human cells lack the biochemical machinery to synthesize phenylalanine, tryptophan, and tyrosine de novo, these aromatic amino acids are obtained from the breakdown of proteins and peptides by bacteria in the colon (Figure 4). The availability of these amino acids is dependent on the presence of bacterial species that can synthesize them, as well as the peptide substrates (including, but not limited to, β-casein and its breakdown products) that are required for their synthesis.^105^ As several key neurotransmitters and neurohormones are synthesized from the aromatic amino acids (eg, dopamine, adrenaline, noradrenaline, serotonin, and melatonin), brain function can potentially be affected by a reduced availability of these substrates (Figure 4).

In addition to the aromatic amino acids, the brain is dependent on SCFAs produced by the gut microbiome, and, in turn, the production of SCFAs is influenced by dietary β-caseins, providing a direct link between milk consumption and brain function. SCFAs are fermentation products of colonic bacteria^106^ that possess anti-inflammatory activity^93^ and may improve the function of colonic cells.^107^ Moreover, they are important substrates for neurotransmitter biosynthesis (Figure 4). For instance, acetate serves as an energy source for neurons and astrocytes, as well as acting as a precursor for the synthesis of the neurotransmitters glutamate and γ-aminobutyric acid (GABA).^108^ Propionate and butyrate are involved in the regulation of serotonin synthesis and in the control of the rate-limiting step for the synthesis of dopamine, adrenaline, and noradrenaline (Figure 4).^109^

A recent study in healthy adults showed that the daily consumption of 250 mL of conventional milk for 4 weeks had no effect on overall gut microbial richness or diversity, or on the levels of fecal SCFAs.^110^ In contrast, studies of lactose-intolerant but otherwise healthy individuals^67^^,^^74^ reported that the consumption of conventional milk resulted in lower fecal concentrations of acetate, butyrate, and total SCFAs when compared with the consumption of A1 β-casein–free milk. These findings in human trials are consistent with those of Guantario et al,^82^ who fed 20-month-old BALB/c mice for 4 weeks with a diet supplemented with either conventional milk, A1 β-casein–free milk, or a control diet without milk. Analysis of the fecal material revealed significant differences in the microbial composition between the groups. Additionally, both groups that received milk had an increase in the production of SCFAs, with the mice that received A1 β-casein–free milk having the highest fecal content of SCFAs, particularly of isobutyrate.^82^

These findings suggest that the composition of β-caseins in milk can influence the diversity of gut microbiota and the rate of production of metabolites such as the aromatic amino acids and SFCAs. Since these metabolites are essential for both gut and brain health,^109^ a better understanding of how the β-casein composition of milk can affect the gut microbiota may be important for the maintenance of optimal brain function.

## CONCLUSION

The present review has examined the functional interrelationships between the consumption of milk proteins, β-casein, and their effect on the brain, immune system, and the gut, which together comprise the GBA. Several of the differential effects observed between conventional milk and A1-type β-casein–free milk are likely due to the production of BCM-7, which can influence the CNS via opioid receptors, and which has been linked to CNS disorders. Moreover, different β-casein compositions of milk have a significant effect on gut motility, the species of gut microbes that make up the microbiota, and their metabolites. These changes can influence gut health and inflammation through the generation of important metabolites, such as the aromatic amino acids and SFCAs, which influence brain function and development through their role as substrates for neurotransmitter production. Further research is needed to fully uncover the molecular pathways that link the digestion of β-caseins and BCM-7 to changes in cognitive performance, and more needs to be learned about the basis of the variability between individuals in their responses to β-casein. Insights from precision nutrition have the potential to explain some of the observed variability and may open a pathway towards personalized milk nutrition^111^^,^^112^; however, further research is needed. Nevertheless, this review indicates that the β-casein composition of milk is an important contributor to the effects of milk, and potentially other milk products, on the GBA.
